# Epitope mapping and conservation of antigens from the Multi-Cruzi immunoassay platform

**DOI:** 10.1007/s00430-026-00877-z

**Published:** 2026-05-22

**Authors:** Eric Dumonteil, Claudia Herrera

**Affiliations:** Department of Tropical Medicine and Infectious disease, Celia Scott Weatherhead School of Public Health and Tropical Medicine, and Vector-Borne and Infectious Disease Research Center, Tulane University, 1440 Canal St., New Orleans, LA 70112 USA

**Keywords:** Chagas disease, *Trypanosoma cruzi*, Biomarker, Antibody, Diagnostic, Antigen, Epitope

## Abstract

Infection with the protozoan parasite *Trypanosoma cruzi* can lead to Chagas disease, and diagnosis of infection mostly relies on serological testing, although no gold standard exists. The evaluation of treatment efficacy is also challenging as seronegativization takes decades to be detected. The Multi-Cruzi platform, which provides a serological profile against 15 parasite antigens, has been proposed as promising alternative for confirmatory diagnostic and monitoring of treatment response. To further evaluate this platform, we performed epitope mapping of the Multi-Cruzi antigens with peptide microarrays, using confirmed *T. cruzi* positive samples, as well as serologically discordant samples with positive *T. cruzi* PCR. Epitope conservation among parasite strains was also assessed. We identified multiple linear epitopes among Multi-Cruzi antigens with samples from patients with confirmed serology, although some redundancy in epitopes may limit the breadth of the antibody profile evaluated. On the other hand, this antigen panel showed very limited reactivity with serodiscordant samples with confirmed *T. cruzi* infection, with weaker recognition of fewer epitopes. Epitope conservation ranged from highly conserved to more variable. The usefulness of this platform may be limited to a fraction of patients and parasite strains. The addition of alternative antigens may help improve the monitoring of treatment response of serodiscordant patients.

## Background

Infection with the protozoan parasite *Trypanosoma cruzi* can lead to Chagas disease, characterized by chronic cardiac and/or digestive disease in 30–40% of cases, while the majority may remain asymptomatic [1]. With at least 10 million cases [2], and an annual economic burden of over $30 billion in Latin America [3], it is one of the most important parasitic diseases of the Americas, although it is highly neglected. Diagnosis of infection occurs mostly during the chronic phase and relies on serological testing to detect antibodies against the parasite. However, no gold standard exists, and the performance of current serological tests can vary significantly depending on the population tested and assay [4–7]. Thus, there are growing efforts to identify new antigens and develop new tests with enhanced performance for the accurate detection of cases [8–14]. This is complicated by extensive *T. cruzi* genetic diversity, as the parasite is divided into seven major clades referred to as discrete typing units (DTUs) named TcI to TcVI and TcBat [15]. This diversity is thought to affect diagnosis as well as disease progression, although clear associations have been difficult to uncover [4, 16].

Following drug treatment of patients with Benznidazole or Nifurtimox, there is also a critical need for tests of cure. Indeed, the current gold standard is seronegativization, but it may take decades to occur after the end of treatment, making it of limited clinical use, and the evaluation of treatment efficacy remains challenging [17–20]. Thus, early indicators of cure are needed and various biomarkers have been evaluated [21, 22]. Among these, a decrease in antibody titer against specific parasite proteins has been proposed [23–26].

One of the recently developed platforms is the Multi-Cruzi assay, which is emerging as a promising alternative for both the diagnosis of *T. cruzi* infection and the monitoring of drug treatment efficacy. It includes a panel of well characterized parasite antigens, similar to those used in several commercial ELISA assays [11], but with the key advantage of providing binding data to individual antigens. The assay was initially designed for confirmatory testing of *T. cruzi* infection and included 12 *T. cruzi* diagnostic antigens [27]. It was then expanded to include 16 antigens, including DTU-specific epitopes from the TSSA antigen, and serological profile against these was evaluated for monitoring therapeutic response in a mouse model of *T. cruzi* infection [28]. The response of children to treatment has also been evaluated, and a progressive and sustainable decline in antigen reactivity was detected with the Multi-Cruzi platform, predicting seroreversion much earlier than conventional serology and indicative of parasitological cure [29]. These promising results were recently expanded to the evaluation of treatment efficacy in adult patients, evidencing an antibody decline against Multi-Cruzi antigens allowing to assess treatment efficacy after just 6 months of follow-up post-treatment [30].

To further evaluate the Multi-Cruzi platform, we investigated here the nature of the epitopes recognized by human plasma/sera and evaluated their reactivity using a panel of well characterized samples that included confirmed *T. cruzi* positive samples, as well as serologically discordant samples that are often discarded from analyses. The sequence conservation of these epitopes was also assessed among a broad range of parasite strains.

## Methods

### Patient samples

Human plasma consisted of archived de-identified samples from a previous study on congenital *T. cruzi* transmission [31] that had been extensively characterized by serological and molecular assays. Briefly, these included maternal venous blood/plasma samples collected at birth in Argentina (*N* = 11), Honduras (*N* = 10) and Mexico (*N* = 10). None of the mothers had been treated previously for *T. cruzi* infection. These samples had been tested with Chagas STAT PAK^®^ (Chembio Diagnostic, Inc.), *Trypanosoma* Detect™ (InBios), Chagatest ELISA recombinant v.3.0 (Wiener), and Hemagen^®^ Chagas’ Kit (Hemagen) as well as by quantitative real time PCR and two end-point PCR assays, one targeting nuclear satellite DNA (primers TcZ1-TcZ2) [32], and one targeting minicircle DNA (primers 121–122) [33], with extensive quality control and cross-validation among participating laboratories [4, 31]. *Trypansoma cruzi* genotypification of some of these samples showed that DTU distribution was similar among infected mothers from the three countries, with a high frequency of TcII-TcV-TcVI DTUs, and mixed infections with TcI [34]. On the other hand, reactivity in *T. cruzi* diagnostic tests varied greatly among samples, with many presenting discordant serology among tests [4, 10, 31]. Samples were stored at -80 °C, with constant temperature monitoring, and serological testing with *Trypanosoma* detect and Chagatest ELISA was performed again prior use in this work to ensure optimal sample preservation. For microarray analysis, IgG antibodies were purified from plasma samples using Thermo Scientific™ Melon™ Gel IgG Spin Purification Kit, per kit instructions, and concentration was measured on a Nanodrop2000 spectrophotometer.

### High-density peptide microarrays for epitope mapping

Sequences from 15 *T. cruzi* antigens included into the Multi-Cruzi assay (Table 1) were used to generate 15-mers peptides with an overlap of 13 amino acids, which were evaluated in high density peptide microarrays as described before [10]. The arrays also included peptides from the CMV large phosphoprotein antigen and a Herpes envelope epitope, as well as Cy3 blank spot as technical controls [10]. Microarrays were incubated with pools of purified IgG for epitope mapping of *T. cruzi* antigens to assess potential differences in epitopes according to previous serological testing. Thus, this included a pool of unequivocally positive samples, i.e. positives for > 3 serological tests and *T. cruzi* PCR (*N* = 9), and a pool of unequivocally negative samples, i.e. negative for all serological and PCR assays (*N* = 11). We also used a pool of serodiscordant samples, typically reactive in only one of the serological assays, but positive by PCR (*N* = 11). Each pool included patient samples from Argentina, Honduras and Mexico (*N* = 3–5 from each country). Fluorescent signal intensity and Z scores were reported for IgG binding to individual peptides in the microarrays [10]. Microarray data are available in NCBI GEO database, accession #GSE235074.

**Table 1:** *T. cruzi* antigens

Antigen	Accession/reference	Length*	15-mers tested^#^
CRA	Q26947	1128	562
Surface antigen-2	Q4DGM0	1254	386
MAP	Q4D5A7	1091	539
TcD	Q4E0M7	1133	560
SAPA	CAA40511	879	433
TcR39	Q7M3R5	321	154
60 S rib. prot. L-19	Q4E2W4	372	179
Trans-sialidase	Q4E0B0	898	442
KMP-11	Q9U6Z1	92	39
Tc40	Q26872	915	451
TcR69	Q7M3W1	97	42
Cruzipain	P25779	467	227
Tc24-C4	[50]	211	100
TSSA TcII/V/VI	XP_808931	92	39
TSSA DTU peptides	[13]	15	14

*Length is given as number of amino acids in primary sequence

# The antigens were divided into 15-mers peptides with 13 amino acid overlap, the total number of peptides covering the full-length sequence is indicated

### Identification of epitopes

Heatmaps of IgG reactivity along each antigen sequence were generated and compared for negative, positive, and discordant sample pools to identify epitopes. Because many of the antigens used consist of repeated sequences/epitopes, sequence alignments of the reactive epitopes were constructed to assess epitope conservation along each antigen, which was visualized with WebLogo [35]. The reactivity of individual epitopes was averaged over their repeats.

### Epitope conservation among DTUs

To assess epitope conservation among multiple parasite strains and DTUs, BLASTp searches were performed on a custom database from 32 *T. cruzi* genomes from multiple countries and covering all DTUs (TcI: 12; TcII: 4; TcIII: 3; TcIV: 5; TcV: 3 and TcVI: 5; Table 2). Sequence alignments of epitopes were constructed and visualized using WebLogo as described above to assess conservation among strains. The number of occurrences of the epitope per genome was calculated, as well as the proportion of genomes from each DTU in which epitopes were identified.

**Table 2:** *T cruzi* strains analyzed

Strain	DTU	Country	Host	Source*
Arequipa	TcI	Peru	Human	GCA_003594685.1
Brazil_A4	TcI	Brazil	Rat	GCA_015033625.1
CGl14	TcI	Colombia	Human	SRX1851527
Corpus_Christi	TcI	USA	Human	SRX1054555
Dm28c	TcI	Colombia	Opossum	GCA_003177105.1
G_strain	TcI	Brazil	Opossum	GCA_003719455.1
H1Yuc	TcI	Mexico	Human	SRX1851500
Silvio_X10	TcI	Brazil	Human	GCA_000188675.2
TBM3324	TcI	Ecuador	Triatomine	SRR3676267
TcLM58	TcI	USA	Macaque	Unpublished
TD25	TcI	USA	Triatomine	SRR3676273
WB1	TcI	USA	Triatomine	Unpublished
Berenice	TcII	Brazil	Human	GCA_013358655.1
Esmeraldo	TcII	Brazil	Human	GCA_000327425.1
Ycl2	TcII	Brazil	Human	GCA_003594485.1
Ycl6	TcII	Brazil	Human	GCA_015033655.1
Ikiakarora	TcIII	Colombia	Triatomine	GCA_010117215.1
Tc231	TcIII	Brazil	Human	GCA_900252365.1
M6241	TcIII	Brazil	Human	SRR4023054, SRR4023055
CanIII	TcIV	Brazil	Human	SRR1996498, SRR1996501
Sum4Cl2	TcIV	Mexico		Unpublished
Sum4Cl3	TcIV	Mexico		Unpublished
TcGI52	TcIV	USA	Macaque	Unpublished
TcMD12	TcIV	USA	Macaque	Unpublished
9280cl2	TcV	Bolivia	Human	SRR1996492, SRR1996493, SRR1996496, SRR1996497, SRR1996502
HE	TcV	Argentina	Human	Unpublished
SC43	TcV	Bolivia	triatoma	GCA_015455285.1
CL	TcVI	Brazil	Human	GCA_003719155.1
CL_Brener	TcVI	Brazil	Human	GCA_000209065.1
H1_Panama	TcVI	Panama	Human	Unpublished
TCC	TcVI	Chile	Human	GCA_003177095.1
VD	TcVI	Argentina	Human	Unpublished

*Accession number of genome assemblies (NCBI genomes) or raw sequences (NCBI SRA) are indicated

### Statistical analysis

Antibody levels expressed as optical density (OD) readings and blood parasite burden are presented for individual samples, and compared using Student t test. Z scores for IgG binding intensity to individual peptides were calculated for comparison among groups.

## Results

**Fig. 1:**
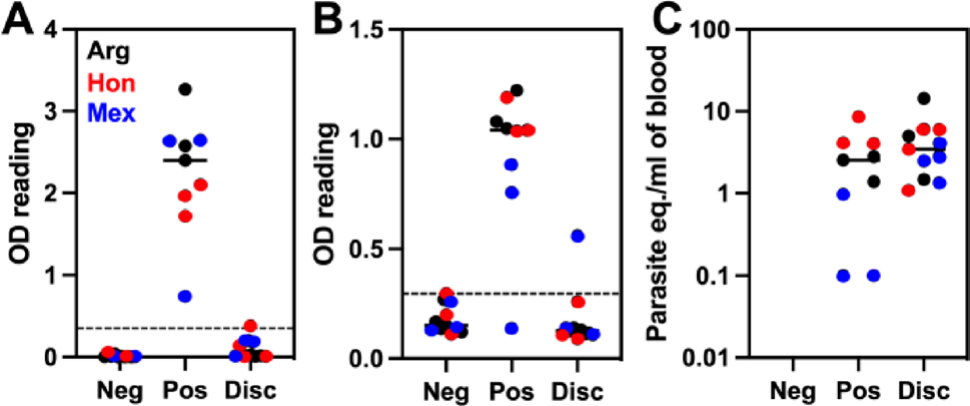
Serological and molecular characterization of plasma samples. Individual plasma samples reactivity with Wiener Chagatest ELISA (**A**), Hemagen Chagas ELISA (**B**) and parasite burden measured by qPCR (**C**). Samples were classified as negative (Neg), positive (Pos), or discordant (Disc) based on serological and molecular assays and are color-coded based on country of origin. There was no significant difference in parasite burden between positive and discordant samples (*t* = 1.09; *P* = 0.29). The dotted line in A and B indicates the cut-off for reactive samples

Epitope mapping was performed using patient samples from *T. cruzi* negative subjects, confirmed positives, and serodiscordant but *T. cruzi* positive samples, respectively. As shown in Fig. 1. There were no significant differences in parasite burden between positive and serodiscordant samples (*t* = 1.09; *P* = 0.29), although their reactivity in ELISA assays was very different, with discordant samples presenting no/low reactivity in Wiener and Hemagen ELISAs.

**Fig. 2:**
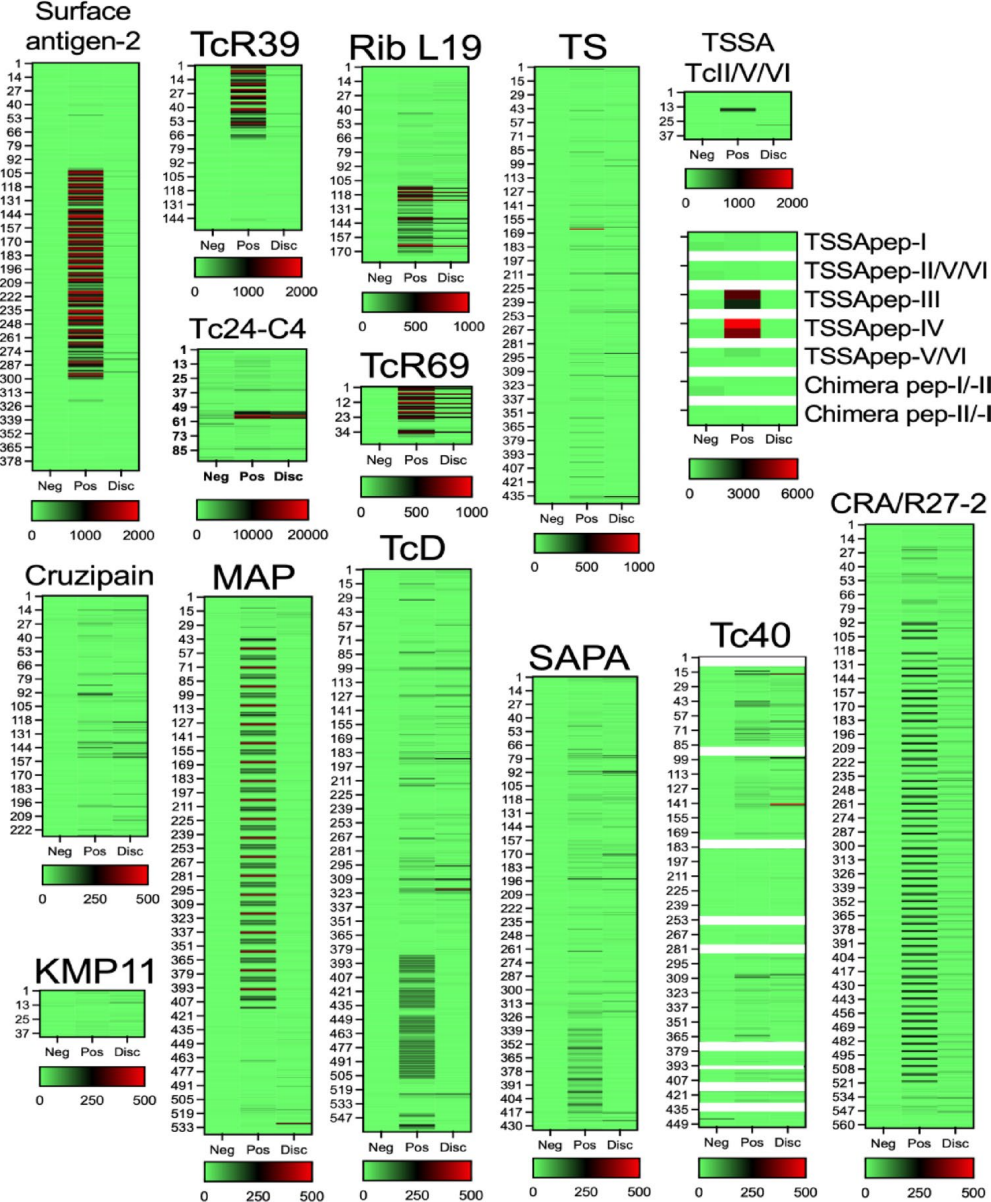
Epitope mapping of *T. cruzi* antigens. Heatmaps of plasma sample reactivity against overlapping peptides covering the indicated antigens in microarrays. Peptides are numbered from the top of heatmaps, corresponding to peptides from the amino terminal to the bottom, corresponding to the carboxyl terminal. Samples pools were negative (Neg), positive (Pos), or discordant (Disc) based on serological and molecular assays for *T. cruzi*. Note differences in color scales among heatmaps

We then used these plasma pools for epitope mapping of the Multi-Cruzi antigens in peptide microarrays using purified IgG. As expected, the pool of negative samples showed no/limited reactivity against these antigens, although a somewhat higher background was observed with Tc24 antigen, suggesting an overall excellent specificity. On the other hand, the pool of positive samples exhibited strong reactivity to multiple epitopes from all antigens, although the intensity of reactivity varied across epitopes and antigens (Fig. 2). The strongest reactivity, targeting multiple epitopes, was detected along Surface antigen-2 and TcR39, as well as for a single epitope of Tc24-C4 antigen. For the TSSA antigen, which has been proposed for parasite DTU-specific serology [13, 36], a conserved epitope was recognized, together with some reactivity for a DTU-specific epitope corresponding to TcIII and TcIV DTUs, and no reactivity was detected for TcI and TcII/VI TSSA epitopes or TcI/TcII chimeric epitopes. Reactivity to strings of repeated epitopes was observed along L-19, TcR69, MAP, TcD, SAPA, Tc40 and CRA. Finally, cruzipain, KMP-11 and Tc40 presented the weakest reactivity to a limited number of epitopes. Remarkably, the pool of serodiscordant but *T. cruzi* positive samples presented a reactivity pattern strikingly different from that of the positive pool, with very limited reactivity to the epitopes from most antigens tested (Fig. 2). Indeed, only the Tc24-C4 antigen presented a comparable reactivity for the positive and serodiscordant pools, targeting a single epitope region. The serodiscordant pool presented a weak reactivity to epitopes from L-19 and TcR69 antigens, and reactivity was even weaker for all remaining antigens (Fig. 2).

**Fig. 3:**
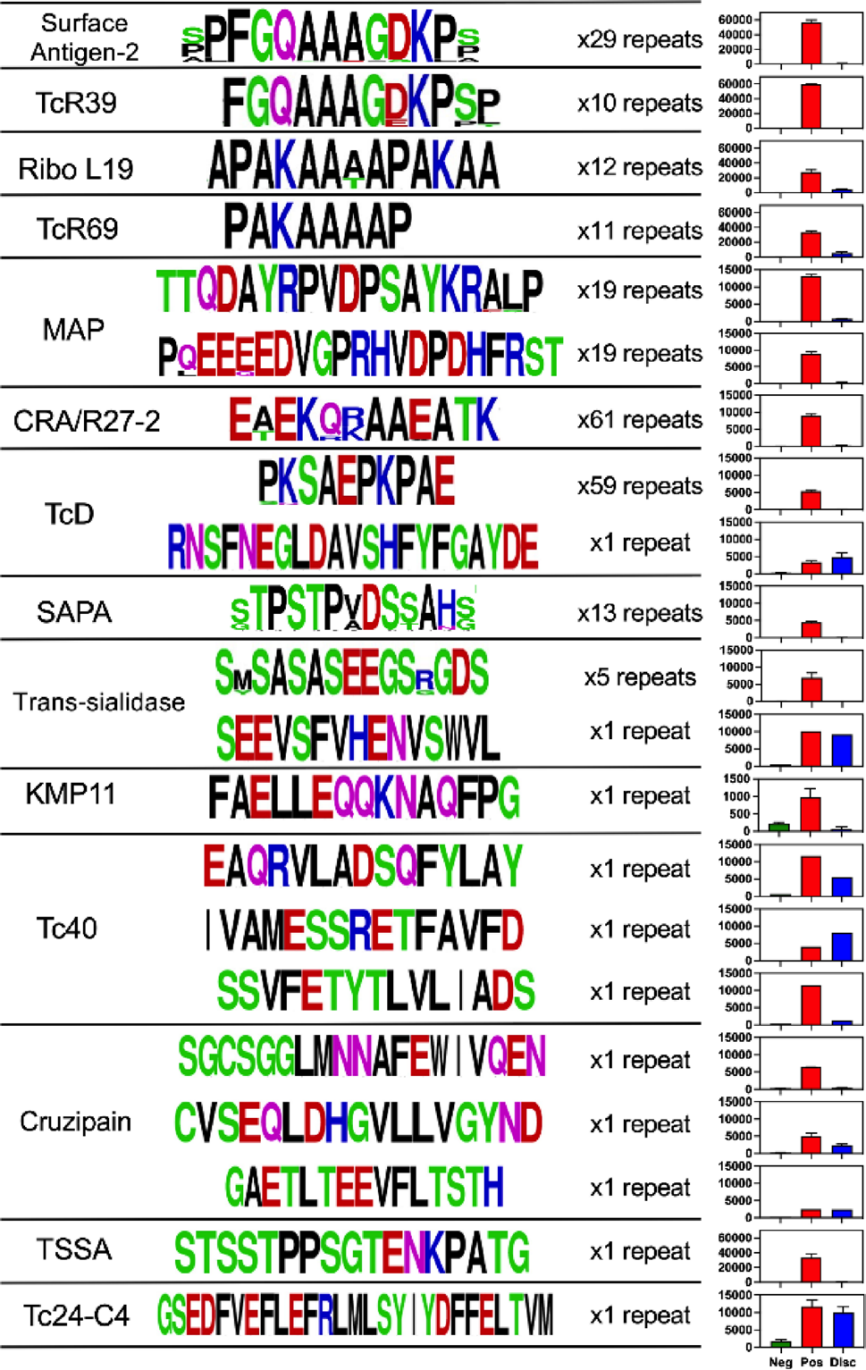
Epitope sequences and reactivity. Sequences of the main epitopes from the indicated antigens are shown, using WebLogo to show their conservation among repeats within each antigen. Amino acids are color-coded according to their chemistry: Green= Polar; Purple= Neutral; Blue= Basic; Red= Acidic; Black= Hydrophobic. The number of repeats of each epitope within the antigen sequence is indicated, as well as their reactivity with negative (Neg), positive (Pos) and discordant (Disc) pools of IgGs. Note the differences in scale on the bar graphs

We then assessed the sequence diversity of the epitopes, which comprised a total of 21 highly reactive sequences (Fig. 3). The reactive epitopes from several antigens consisted of repeated sequence motifs with a high level of conservation across motifs as noted before [37–39]. This is for example the case for the epitopes from Surface antigen-2, TcR39, Ribo L-19, TcR69, MAP, CRA, TcD, SAPA and trans-sialidase, which dominant epitope was repeated 5–61 times within these proteins (Fig. 3). Furthermore, epitopes from Surface antigen-2 and TcR39 were identical, as well as those from L-19 and TcR69 antigens. Overall, this redundancy reduced the diversity of epitopes of the Multi-Cruzi platform to 19 major epitopes. All other antigens, namely KMP-11, Tc40, cruzipain, TSSA and Tc24-C4 presented a limited number of unique epitopes. The reactivity of all epitopes was strong with the positive pool of samples, but negligible for all repeated epitopes with the pool of discordant samples. Only a few epitopes from TcD, trans-sialidase, Tc40 and Tc24-C4 showed some reactivity with this discordant sample pool. Together, these results indicate that these antigens/epitopes are highly reactive and specific for positive control samples but have a very limited reactivity with discordant but *T. cruzi* positive samples.

**Fig. 4:**
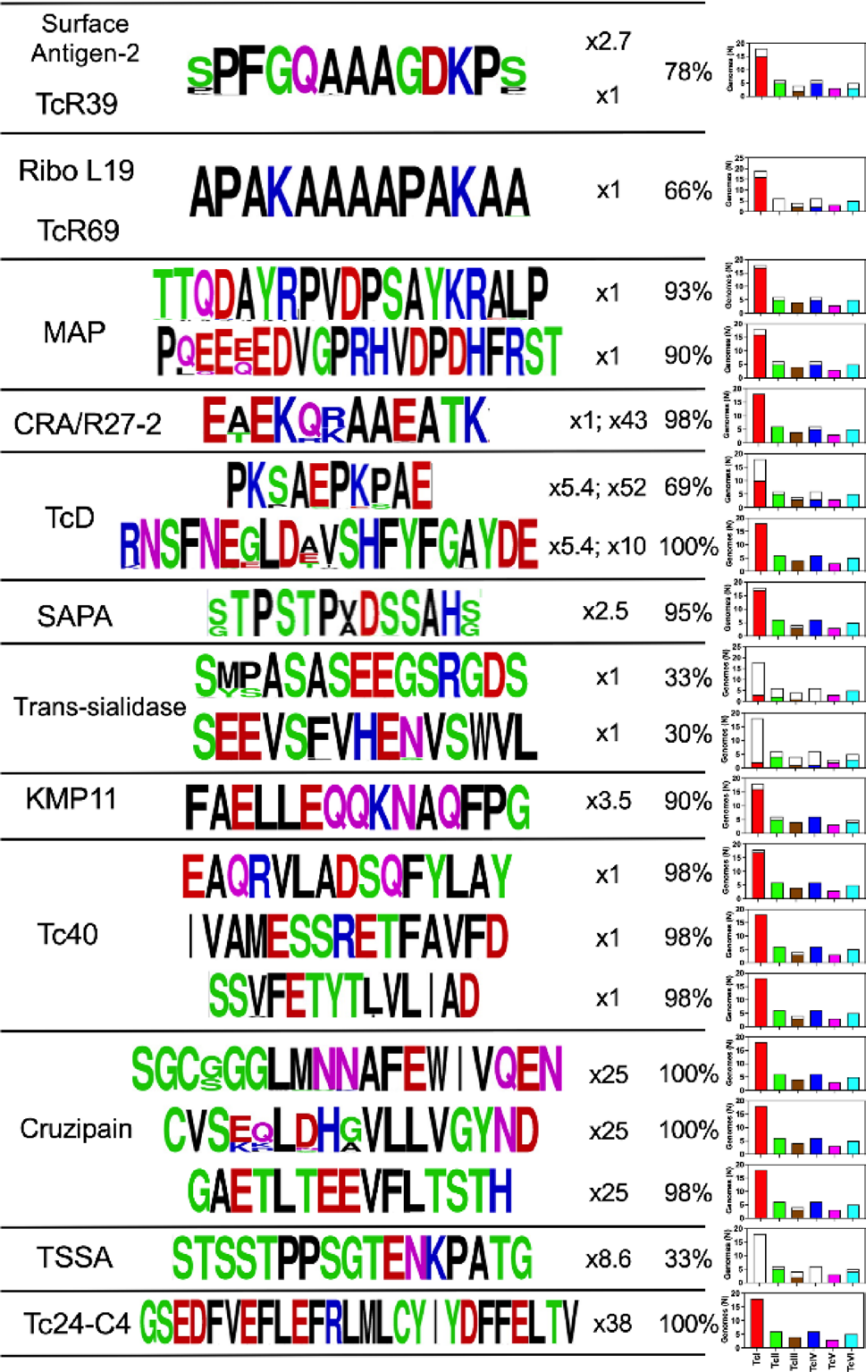
Epitope sequence conservation among *T. cruzi* genomes. The sequence of the main epitopes from the indicated antigens is shown, using WebLogo to show their conservation among parasite genomes. Amino acids are color-coded according to their chemistry: Green= Polar; Purple= Neutral; Blue= Basic; Red= Acidic; Black= Hydrophobic. The average copy number of each antigen within individual genomes is indicated, together with the copy number of the epitope if present in other proteins (for CRA/R27-2 and TcD). The percentage of genomes in which the epitope is predicted to be present is also indicated. The bar graphs present details of DTU distribution of the epitopes, with the colored bar corresponding to the number of genomes in which epitopes are predicted to be present, and the empty bar genomes in which the epitopes are absent, respectively

Epitope sequence conservation was further assessed among 32 *T. cruzi* strains covering six DTUs. Overall, all epitopes were highly conserved, with only a few amino acid positions presenting some variability within their sequences (Fig. 4). Many epitopes were also derived from multicopy genes, and were thus present in multiple copies within genomes, in addition to some of them being repeated withing single antigens. Thus, while cruzipain or Tc24-C4 epitopes were not repeated in the sequence, these antigens had an average 25 or 38 copies per genome, respectively. Some epitopes, such as those from CRA and TcD were also present in several other proteins increasing their potential copy number.

Regarding the distribution of epitopes among DTUs, most (13/19, 68%) were present across all six DTUs and over 90% of the strains. Nonetheless, six epitopes were not extensively represented among DTUs. For example, the two trans-sialidase epitopes were only identified in one-third of the strains, and were absent from a large proportion of TcI, TcIII, and TcIV strains, while being present in most TcII, TcV and TcVI strains (Fig. 4). Similarly, the dominant epitope APAKAAAAPAKAA from Riboprotein L19 and TcR69 antigens was only detected in 66% of strains, being absent from all TcII strains and from some of the TcI, TcIII and TcIV strains. The major TSSA epitope was also only present in TcII, TcIII, TcV and TcVI, and absent from TcI and TcIV, confirming some of its DTU specificity.

## Discussion

The Multi-Cruzi assay is emerging as a promising platform, particularly for the monitoring of the response to drug treatment of Chagas disease patients, as it may provide early indication of treatment efficacy [29, 30]. It thus warrants further evaluation of the reactivity against the parasite antigens included in this assay. Importantly, many *T. cruzi* infected patients present discordant serology, which may affect assay performance. Indeed, as clearly shown with the samples from our panel, some patients have a low/absent serological response to parasite antigens included in several commercial ELISA tests, but a comparable *T. cruzi* parasite burden as that of well confirmed seropositive patients [4]. Thus, we performed here a detailed epitope mapping of these antigens, using well characterized plasma samples.

Epitope mapping with our seropositive sample pool largely confirmed the reactivity of all evaluated antigens, although with different intensity. Furthermore, many of the specific reactive epitopes detected in our study are also identical/highly similar to the epitopes previously identified in these antigens. This is for example the case for Surface antigen-2 and TcR39 [40–42], L-19 and TcR69 [40–42], TSSA [43, 44], CRA [45], SAPA [46, 47] and KMP-11 [48] epitopes. Importantly, some of these epitopes were redundant as Surface antigen-2 and TcR39 included the same epitope, as well as L-19 and TcR69 antigens. Thus, antibody recognition of these antigens can be expected to be highly correlated, and this redundancy reduces the diversity of the serological profile evaluated with the Multi-Cruzi assay, which was designed to assess antibody profile against multiple antigens. On the other hand, several of the antigens included multiple repeats of their epitopes, likely providing increased sensitivity for antibody reactivity. Indeed, these epitopes have been identified as immunodominant based on their strong reactivity with patient sera in multiple studies [11, 42].

On the other hand, reactivity against these antigens was strikingly absent or very low with the pool of serodiscordant samples, indicating a very different serological profile of these patients. The only epitope with comparable reactivity between the positive and discordant pools was from Tc24-C4. Remarkably, all dominant and multicopy epitopes reactive with the positive pool showed no or limited reactivity with serodiscordant samples. A few alternative epitopes were reactive with these discordant samples, but intensity did not reach that of the positive sample pool. This is in agreement with the very different antibody profile from these patients with discordant serology and their lack of antibody in commercial ELISA assays [4, 10]. Importantly, these discordant samples included patients from Mexico, Honduras and Argentina, indicating a broad geographic distribution of these patients, which may represent an important fraction of patients.

Epitope sequence conservation among parasite strains was generally high, with only a few variable amino acid positions within each epitope, suggesting that these should be broadly immunogenic for infections with any parasite DTU, at least in non-serodiscordant patients. Nonetheless, some parasite strain/DTU restriction was observed for some of the epitopes, notably from the trans-sialidase, which epitopes were presents in TcV and TcVI, but rarely in other DTUs and for the immunodominant epitope from riboprotein L-19/TcR69, which was absent from TcII, and also missing from some TcIII and TcIV strains. This suggests that infections with some strains/DTUs may not induce antibodies against these antigens.

A limitation of our study is that epitope mapping was performed with pools of samples, masking individual variability in antigen/epitope recognition. Indeed, there is growing evidence that the antibody profile of *T. cruzi* infected patients is highly variable among individuals [10, 49], making the identification of an antigen panel recognized by all or most patients challenging. Thus, futher studies may explore interindividual variability in epitope recognition profile as well as potential geographic differences. Increasing sample size may also allow to identify additional epitopes, although these would only be recognized by a smaller proportion of infected patients, and thus poorly informative.

In conclusion, our results confirmed the good reactivity of the Multi-Cruzi antigens with IgG samples from Chagas disease patients with confirmed serology, and the high level of sequence conservation of the identified epitopes. The redundancy of some epitopes may limit the breadth of the antibody profile evaluated, as well as the absence of some of the epitopes from some parasite strains and DTUs. In addition, this antigen panel showed limited reactivity with serodiscordant samples with confirmed *T. cruzi* infection, with weaker recognition of fewer epitopes. This may restrict the usefulness of this platform to a fraction of patients and alternative antigen panels would be needed for the monitoring of antibody profiles in all Chagas disease patients.

## Data Availability

Microarray data are available in NCBI GEO database, accession #GSE235074.
